# Unexplained Hypokalemia in a Patient With Obesity Harboring an Armadillo Repeat-Containing 5 (ARMC5) Gene Variant: A Case Report

**DOI:** 10.7759/cureus.79326

**Published:** 2025-02-19

**Authors:** Akihiko Taguchi, Shinsuke Uraki, Masaru Akiyama, Yuko Nagao, Kenji Watanabe, Yoichi Mizukami, Yasuharu Ohta

**Affiliations:** 1 Department of Endocrinology, Metabolism, Hematological Science and Therapeutics, Graduate School of Medicine, Yamaguchi University, Ube, JPN; 2 Department of Diabetes, Endocrinology and Metabolism, Horiguchi Memorial Hospital, Wakayama, JPN; 3 First Department of Internal Medicine, Wakayama Medical University, Wakayama, JPN; 4 Health Science Center, Yamaguchi University, Ube, JPN; 5 Institute of Gene Research, Science Research Center, Yamaguchi University, Ube, JPN

**Keywords:** armc5, case report, hypokalemia, obesity, weakness of the limbs

## Abstract

Hypokalemia of unknown cause can often be challenging to diagnose. Although armadillo repeat-containing 5 (ARMC5) gene mutations are primarily associated with primary bilateral macronodular adrenal hyperplasia and Cushing's syndrome, their potential role in other endocrine disorders remains largely unexplored. A 50-year-old man presented with limb weakness and persistent hypokalemia. Comprehensive screening tests, including imaging and endocrinological evaluations, ruled out primary aldosteronism, Cushing's syndrome, and other common causes of hypokalemia. Genetic analysis revealed a heterozygous ARMC5 variant. As the patient also presented with obesity, and given that previous mouse and in vitro studies suggest possible interactions between ARMC5 and mineralocorticoid pathways, we hypothesize that the mechanism of hypokalemia may involve adipose tissue function. This case describes an association between an ARMC5 variant, unexplained hypokalemia, and obesity. Although a direct causal relationship cannot be established from a single case, the systematic exclusion of common causes and known ARMC5 functions in mineralocorticoid pathways suggest potential mechanistic links. This observation warrants further investigation, particularly through comprehensive screening of electrolyte disorders in patients with ARMC5 variants, analysis of mineralocorticoid activity in adipose tissue samples from these patients, and molecular studies examining ARMC5's direct role in the regulation of mineralocorticoids in adipose tissue.

## Introduction

Hypokalemia is a clinically significant electrolyte disorder that can lead to various cardiovascular and neuromuscular complications. Common etiologies include excessive potassium loss, insufficient potassium intake, and redistribution of potassium in the body [1]. Obesity is associated with several metabolic and hormonal disorders and results in a complex interplay of clinical manifestations, including electrolyte imbalance; however, the relationship between obesity and hypokalemia has not been widely reported. Moreover, a previous study proposed that mineralocorticoid-releasing factors secreted by adipocytes are an intervening factor in the impact of obesity on hypertension [2]; however, the detailed underlying mechanisms are not fully elucidated, and the independent role of obesity in hypokalemia requires further investigation.

Armadillo repeat-containing 5 (ARMC5) gene is primarily associated with primary bilateral macronodular adrenal hyperplasia and Cushing's syndrome [3]. Notably, ARMC5 variants in the African American population are positively correlated with hypertension and aldosterone levels, suggesting their potential involvement in mineralocorticoid signaling [4]. Although these findings suggest potential connections between ARMC5 variants, obesity, and endocrine regulation, the exact mechanisms remain poorly understood. Therefore, we present this case report that explores these relationships and their potential implications for clinical practice, particularly in the context of unexplained hypokalemia in obesity.

## Case presentation

**Figure 1:**
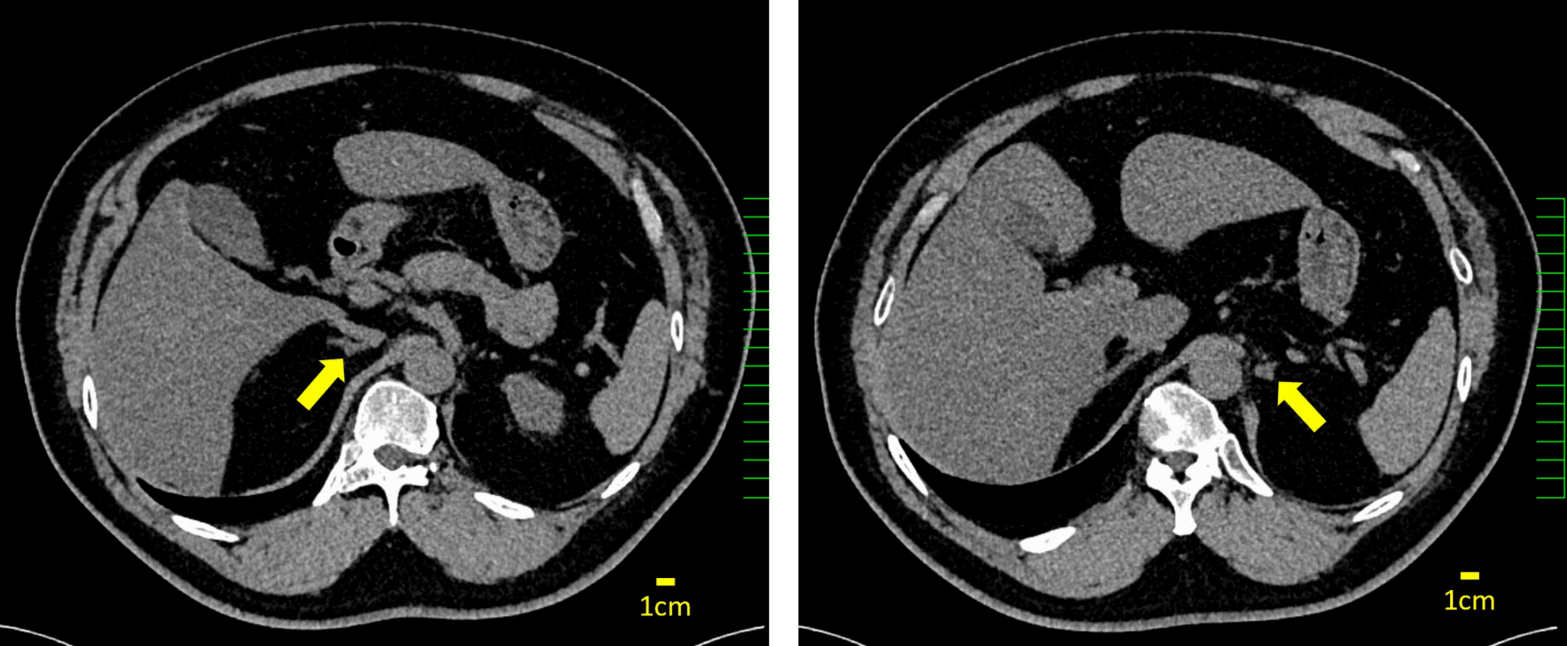
Axial computed tomography scan demonstrating normal adrenal glands. The figure shows the periadrenal area with the adrenal glands marked by yellow arrows. Both adrenal glands measured less than 10 mm in body thickness (normal range for adrenal body: right 7.2±1.8 mm, left 8.8±1.9 mm), with all measurements falling within normal reference ranges [5]. This finding supports the exclusion of adrenal hyperplasia as a contributing factor to hypokalemia.

A 50-year-old Japanese man receiving treatment for hypertension had intermittent muscle weakness over a two-year period that was expected to resolve spontaneously. Three months before presentation, the patient experienced muscle weakness in both lower limbs upon waking up, followed by difficulty moving their body the next day. The patient was admitted to a local hospital with hypertension (142/94 mmHg), hypokalemia (2.2 mmol/L), normal thyroid function, and no adrenal enlargement on computed tomography (CT) (Figure 1). The patient had no history of alcohol or drug abuse, steroid use, oral herbal medication use, or family history of hypokalemia, endocrine disorders, or malignancy. Subsequent treatment involved continued nifedipine and temporarily prescribed potassium chloride extended-release tablets before referral to our clinic for further investigation.

Upon admittance to our hospital, a physical examination revealed a blood pressure of 138/92 mmHg, a pulse rate of 74 beats/min, a body mass index of 41.8 kg/m², a height of 174.9 cm, and a weight of 128 kg. Chest and abdominal examination revealed no abnormal findings and muscle strength was normal at the time of examination. No edema of the lower extremities or Cushing-like features (moon face, purple striae, or hypertrichosis) were observed. Routine laboratory tests revealed extremely low serum potassium (2.9 mmol/L) and increased urinary potassium excretion (115 mmol/24 h) (Table 1). Renal artery stenosis was ruled out through contrast-enhanced CT imaging, which showed no evidence of stenosis. Additionally, the baseline plasma renin activity was 1.1 ng/mL/h (reference range: 0.3-2.9 ng/mL/h), which was in the lower normal range and did not show the elevation typically seen in renal artery stenosis. Furthermore, as shown in Table 2, the captopril challenge test demonstrated no significant changes in renin levels, providing additional confirmation for the exclusion of renal artery stenosis. Urinalysis was normal, and salt-losing tubulointerstitial nephritis was ruled out. Arterial blood gas analysis showed a pH of 7.432, a partial pressure of carbon dioxide (PCO_2_) of 45.7 mmHg, bicarbonate (HCO_3_^-^) of 29.9 mEq/L, and a base excess of 5.2 mEq/L, indicating mild primary metabolic alkalosis with respiratory compensation.

**Table 1:** List of laboratory test results. WBC: White blood cell count; Hb: Hemoglobin; Plt: Platelets; Cre: Creatinine; BUN: Blood urea nitrogen; Na: Sodium; K: Potassium; Ca: Calcium; Mg: Magnesium; Glu: Glucose; TSH: Thyroid-stimulating hormone; FT4: Free thyroxine; ACTH: Adrenocorticotropic hormone

Laboratory items	Measured values	Reference range
WBC (×10⁹/L)	7.06	3.3-8.6
Hb (g/L)	155	137-168
Plt (×10⁹/L)	199	158-348
Cre (µmol/L)	72.5	57.5-94.6
BUN (µmol/L)	3.93	2.86-7.14
Na (mmol/L)	141	138-145
K (mmol/L)	2.9	3.6-4.8
Ca (mmol/L)	2.2	2.2-2.5
Mg (mmol/L)	0.82	0.74-0.99
Glu (mmol/L)	4.77	4.05-6.05
TSH (mIU/L)	1.27	0.61-4.23
FT4 (pmol/L)	0.14	0.09-0.19
Plasma ACTH (pg/mL)	11.2	8.7-61.5
Plasma cortisol (µg/dL)	3.7	4.4-21.1
Plasma renin activity (ng/mL/hr)	1.1	0.3-2.9
Plasma aldosterone (pg/mL)	55.7	4.4-82.1
Urinary K (mmol/24 h)	115	4-82.1
Urinary cortisol (nmol/24 h)	69.6	4.3-176
Urinary aldosterone (pmol/24 h)	22.3	1-19.3
Cortisol before and after ACTH stimulation		
Before (µg/dL)	6	
30 min (µg/dL)	20.9	
60 min (µg/dL)	22.8	
Overnight 1 mg dexamethasone suppression test		
Plasma cortisol (µg/dL)	0.5	

**Table 2:** Captopril challenge test. PRA: Plasma renin activity (reference range: 0.3-2.9 ng/mL/hr); PAC: Plasma aldosterone concentration (reference range: 4.0-82.1 pg/mL); ARR: Aldosterone-renin ratio (reference range: <200)

Time	PRA (ng/mL/hr)	PAC (pg/mL)	ARR
Before	1.2	169	140.8
60 min	1.1	99.7	90.6
90 min	0.9	76.1	85.6

After discontinuation of nifedipine, early-morning resting aldosterone was 55.7 pg/mL (normal range: 4.4-82.1 g/mL) and plasma renin activity was 1.1 ng/mL/h (normal range: 0.3­-2.9 ng/mL/h). These values were within normal ranges, and the captopril challenge test was negative, effectively ruling out primary aldosteronism as a cause of hypokalemia (Table 2) [6].

**Figure 2:**
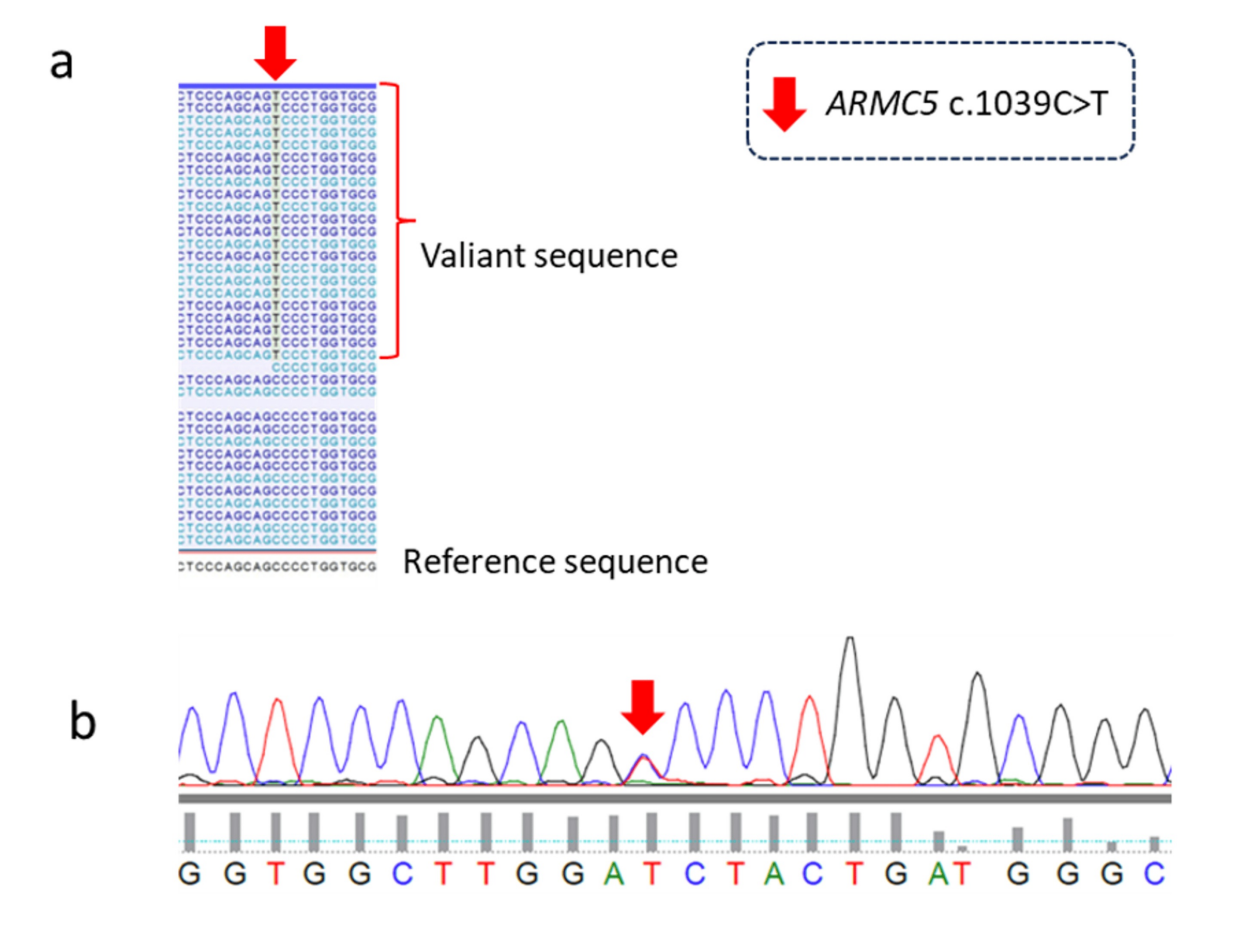
Identification and characterization of the ARMC5 variant in the patient with obesity and hypokalemia. (a) Whole-genome sequencing results showed individual sequencing reads aligned to the genomic region containing the ARMC5 gene. (b) Sanger sequencing chromatogram confirmed the presence of the ARMC5 variant c.1039C>T. ARMC5: Armadillo repeat-containing 5

Given these normal results, further screening for primary aldosteronism was not pursued. Early-morning resting adrenocorticotropic hormone (ACTH) was 11.2 pg/mL and cortisol was 3.7 µg/dL. A rapid ACTH challenge test ruled out hypoadrenalism, and an overnight dexamethasone suppression test showed appropriate cortisol suppression. The complete absence of any clinical features suggestive of hypercortisolism further supported this conclusion. Apparent mineralocorticoid excess syndrome was also considered, but the tetrahydrocortisol/allo-tetrahydrocortisol/tetrahydrocortisone ratio of 1.256 also excluded this diagnosis. Genetic analysis was performed after obtaining patient consent to explore the possibility of other genetic abnormalities causing hypokalemia. Whole-genome analysis of peripheral blood lymphocytes did not identify any genetic abnormalities that could cause generalized hypokalemic periodic paralysis but identified an ARMC5 variant called c.1039C>T. At a coverage depth of 36, the variant allele was observed in 20 reads, whereas the normal allele appeared in 16 reads (Figure 2a). This heterozygous variant was further confirmed by Sanger sequencing (Figure 2b).

**Figure 3:**
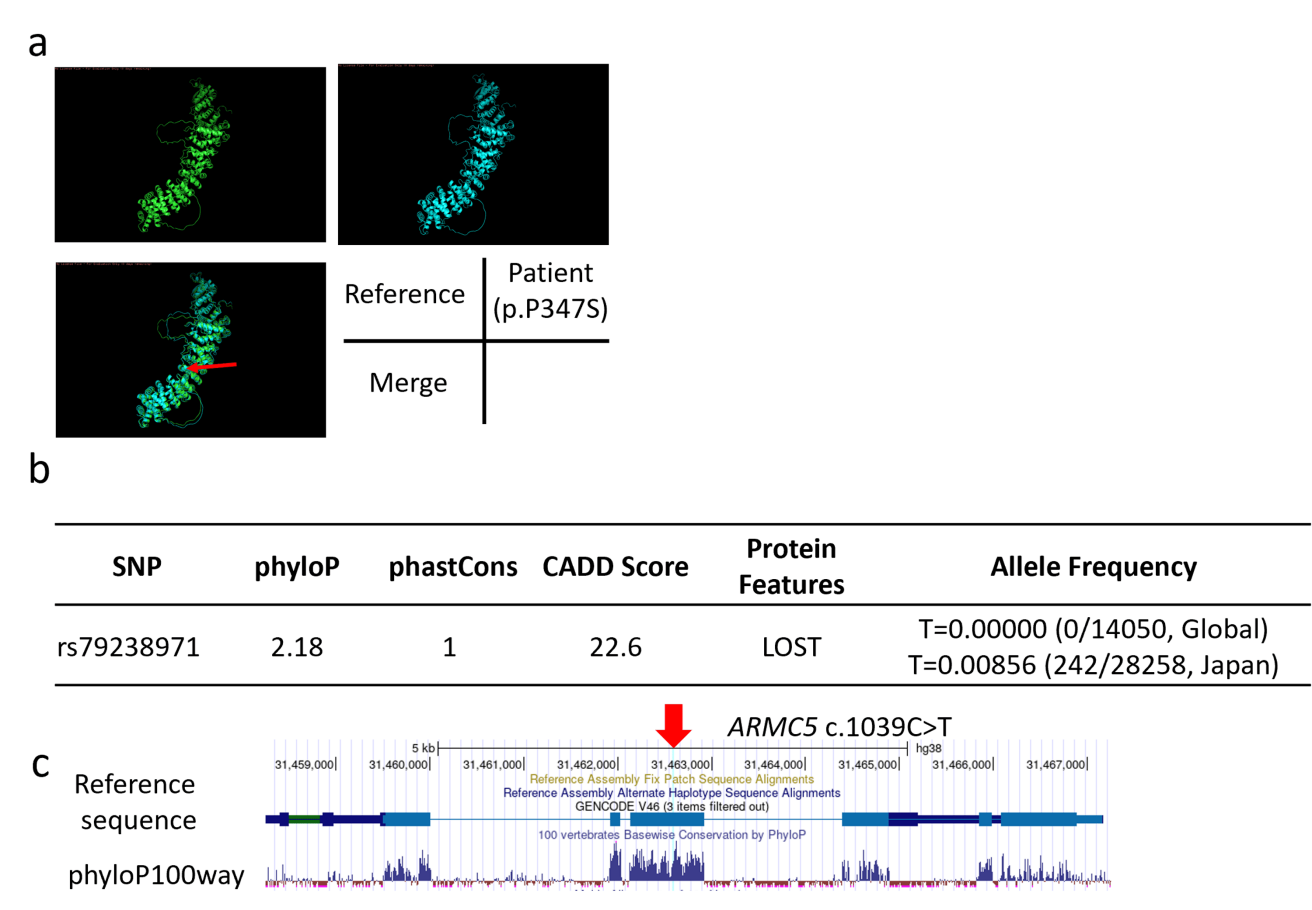
Three-dimensional structures and mutational impact of the ARMC5 protein. (a) Three-dimensional structures of reference and patient ARMC5 proteins simulated and visualized using AlphaFold2 (DeepMind, London, UK) and PyMOL (http://www.pymol.org/pymol). Red arrows indicate mutation sites. (b) SNP number of the variant and protein feature predictions by phyloP, CADD score, and MutationTaster2. Allele frequencies of mutations are based on data from the Allele Frequency Aggregator global project and the Tohoku Medical Megabank project in Japan [11,12]. (c) UCSC Genome Browser (http://genome.ucsc.edu) visualization of the ARMC5 gene, highlighting the location of the variant within a domain that is highly conserved across multiple species, determined by 100-way phyloP. ARMC5: Armadillo repeat-containing 5; SNP: Single nucleotide polymorphism; CADD: Combined Annotation Dependent Depletion; UCSC: University of California Santa Cruz

Three-dimensional protein structures were constructed from the AMRC5 reference and patient sequences (p.P347S) using AlphaFold2 (DeepMind, London, UK) and visualized using PyMOL (http://www.pymol.org/pymol). The mutant constructed from the patient sequence exhibited a structural change (Figure 3a) [7]. Although this variant is registered in dbSNP (rs79238971) and reported as benign in one database, it has not been previously described in clinical cases. When analyzed using Combined Annotation Dependent Depletion (CADD) [8], a tool for predicting virulence, the variant showed high deleteriousness, and the protein was characterized as "LOST" by MutationTaster2 software (Figure 3b) [9]. In addition, the variant exhibited high phyloP and phastCons values and was conserved across species (Figures 3b-3c) [10]. 

The patient is currently receiving potassium chloride extended-release tablets (64 mEq/day). Potassium levels remain at 3.4-3.6 mmol/L. Six months have passed with no onset of muscle weakness, and the patient is satisfied with the current treatment.

## Discussion

This case report describes an instance of unexplained hypokalemia and obesity in a patient with a heterozygous ARMC5 variant. The ARMC5 gene has been primarily linked to primary bilateral macronodular adrenal hyperplasia and Cushing's syndrome, with some studies reporting associations with primary aldosteronism [13].

The late onset of symptoms in the patient at age 50 may be related to the progressive nature of obesity. While the ARMC5 variant was present from birth, the symptoms might not have manifested until adipose tissue accumulation reached a certain threshold. Additionally, age-related metabolic changes may have contributed to the timing of symptom onset.

Obesity appears to play a crucial role in this case. Adipose tissue secretes mineralocorticoid-releasing factors that enhance aldosterone secretion and production, contributing to obesity-induced mineralocorticoid-related hypertension [2,14].

While we initially interpreted the combination of normal plasma aldosterone levels and elevated aldosterone-renin ratio (ARR) as not indicative of primary aldosteronism, it is important to note that the persistently elevated ARR during the captopril challenge test raises some uncertainty about completely excluding this diagnosis. The complexity of this case, with coexisting obesity and the ARMC5 variant, makes it challenging to definitively rule out a mild form of primary aldosteronism. However, the normal plasma aldosterone concentration and the absence of typical imaging findings led us to explore alternative mechanisms, particularly focusing on the potential role of adipose tissue in mineralocorticoid regulation.

In our patient, blood aldosterone levels on screening tests were normal but urinary aldosterone stores were elevated. This discrepancy can be explained by several factors. First, the circadian rhythm of aldosterone secretion, which typically peaks in the early morning due to ACTH action, means that single-point serum measurements may not capture peak levels. Second, mineralocorticoid-releasing factors from adipose tissue may not be secreted at constant rates. Third, the 24-hour urinary measurement provides a better reflection of the overall mineralocorticoid activity compared to a single serum measurement. These factors collectively explain why serum aldosterone levels may appear normal despite evidence of increased mineralocorticoid activity in the 24-hour urine collection. We hypothesize that the hypersecretion of mineralocorticoid-releasing factors occurred outside the early morning hours, independent of ACTH. This hypothesis is supported by the fact that human adipocytes lack ACTH receptors, suggesting a mechanism of ACTH-independent mineralocorticoid-releasing factor secretion from adipose tissue [15].

Recent studies have shown that adipose tissue, similar to adrenal glands, possesses CYP11B, an aldosterone synthase enzyme, and can directly secrete aldosterone [16]. Notably, previous in vitro studies have shown that knockdown of ARMC5 expression in human adrenal cells (H295R) increases aldosterone synthase CYP11B, indicating that a dysfunctional mutation in ARMC5 may increase aldosterone secretion in both adipocytes and adrenal glands [16].

We suggest that the synergistic effect of obesity promotion and increased mineralocorticoid activity, based on the ARMC5 loss-of-function mutation, caused hypokalemia in this case. This hypothesis is supported by the exclusion of other common causes of hypokalemia through comprehensive screening, including whole-genome analysis.

This case has significant clinical implications. Firstly, although ARMC5 variants require a 'second hit' for tumorigenesis, such as in cases of Cushing's syndrome, the heterozygous variants in our patient did not lead to such manifestations. Patients with ARMC5 mutations may be at risk of developing primary bilateral macronodular adrenal hyperplasia, necessitating careful monitoring for signs of adrenal hyperplasia and hypercortisolism [17]. Moreover, the potential role of adipose tissue in mineralocorticoid-releasing factor secretion opens new avenues for studying the pathophysiology of hypokalemia and related endocrine disorders.

Regarding treatment, although potassium supplementation was effective in this case, future studies may explore whether mineralocorticoid receptor antagonists could play a role in managing similar cases [18]. These drugs can inhibit increased mineralocorticoid activity, potentially normalizing potassium levels and alleviating symptoms. Additionally, addressing obesity through diet, exercise, and pharmacotherapy with anti-obesity drugs, such as glucagon-like peptide-1 (GLP1) and glucose-dependent insulinotropic polypeptide-GLP1 receptor agonists, may contribute to correcting hypokalemia.

## Conclusions

In conclusion, this case highlights the potential connection between an ARMC5 variant and unexplained hypokalemia in a patient with obesity. Although a single case cannot establish causation, several observations support a possible mechanistic link. The systematic exclusion of common causes of hypokalemia through comprehensive screening, combined with the established role of ARMC5 in mineralocorticoid regulation and the presence of aldosterone synthase in adipose tissue, provides a plausible biological framework for understanding this association, particularly in the context of obesity.

These findings suggest several important directions for future research. Understanding the prevalence of electrolyte disorders in patients with ARMC5 variants could help establish whether this association occurs more broadly. Moreover, further investigation of mineralocorticoid activity in the adipose tissue of patients with ARMC5 variants, along with molecular studies examining the role of ARMC5 in mineralocorticoid regulation, could illuminate the underlying mechanisms. Such research could provide valuable insights into previously unrecognized pathways of electrolyte homeostasis and identify new therapeutic targets for patients with unexplained electrolyte disorders.
